# Redox regulation by sulfiredoxin-1: bridging cysteine oxidation and liver disease therapeutics

**DOI:** 10.1038/s12276-025-01563-5

**Published:** 2025-10-23

**Authors:** Jong-Won Kim, Mengyun Ke, Donovan Whitfield, Bin Yang, Gu Seob Roh, Wen Xie

**Affiliations:** 1 Center for Pharmacogenetics and Department of Pharmaceutical Sciences, University of Pittsburgh, Pittsburgh, PA USA; 2 Department of Pharmacology, Institute of Medical Sciences, College of Medicine, Gyeongsang National University, Jinju, Republic of Korea; 3 Department of Convergence Medical Science, Gyeongsang National University Graduate School, Jinju, Republic of Korea; 4 Department of Cancer Biology, Lerner Research Institute, Cleveland Clinic, Cleveland, OH USA; 5 Department of Anatomy, College of Medicine, Institute of Medical Science, Gyeongsang National University, Jinju, Republic of Korea; 6 Department of Pharmacology and Chemical Biology, University of Pittsburgh, Pittsburgh, PA USA

**Keywords:** Biochemistry, Liver diseases

## Abstract

Cysteine (Cys) posttranslational modifications play a critical role in regulating protein function, cellular signaling and redox homeostasis in various physiological and pathological conditions. Sulfiredoxin-1 (SRXN1) has emerged as a key regulator of protein redox homeostasis through its involvement in Cys sulfinylation. However, the role of SRXN1 in the pathogenesis of diseases and its therapeutic implications have yet to be fully explored. Beyond its classical function in reactive oxygen species detoxification, SRXN1 also modulates redox-sensitive signaling pathways that govern inflammation, apoptosis and cell survival, making it an essential component of cellular defense against oxidative stress-related damage. Here we highlight the significance of SRXN1 in regulating Cys sulfinylation across a broad spectrum of liver diseases. Furthermore, we emphasize the critical role of SRXN1 in regulating oxidative stress and cellular signaling through its interaction and desulfinylation of target or substrate proteins, both of which are crucial to maintaining cellular function under pathological conditions. Finally, we discuss the potential therapeutic implications of targeting SRXN1 in disease contexts where oxidative stress exacerbates pathological processes. A deeper understanding of SRXN1-mediated redox regulation may offer a novel therapeutic avenue to mitigate Cys oxidation and improve clinical outcomes in various liver disease contexts.

## Sulfiredoxin-1 regulates cysteine in liver disease pathogenesis

Oxidative stress, an imbalance between harmful molecules called reactive oxygen species and the body’s defenses, contributes to many diseases. A key player in managing this stress is a protein called sulfiredoxin 1 (SRXN1). SRXN1 helps to repair proteins damaged by reactive oxygen species, particularly by reversing a process called cysteine sulfinylation, which can impair protein function. This Review explores SRXN1’s role in liver diseases, highlighting its protective effects on hepatocytes under pathological conditions such as acute liver injury, alcoholic liver disease and liver fibrosis. It does this by maintaining redox balance. Researchers used various methods to study SRXN1’s effects, including examining its interactions with other proteins and its impact on cell survival. Results show that, while SRXN1 protects against liver damage, it also aids cancer cell survival in liver cancer.

This summary was initially drafted using artificial intelligence, then revised and fact-checked by the author.

## Introduction

**Fig. 1:**
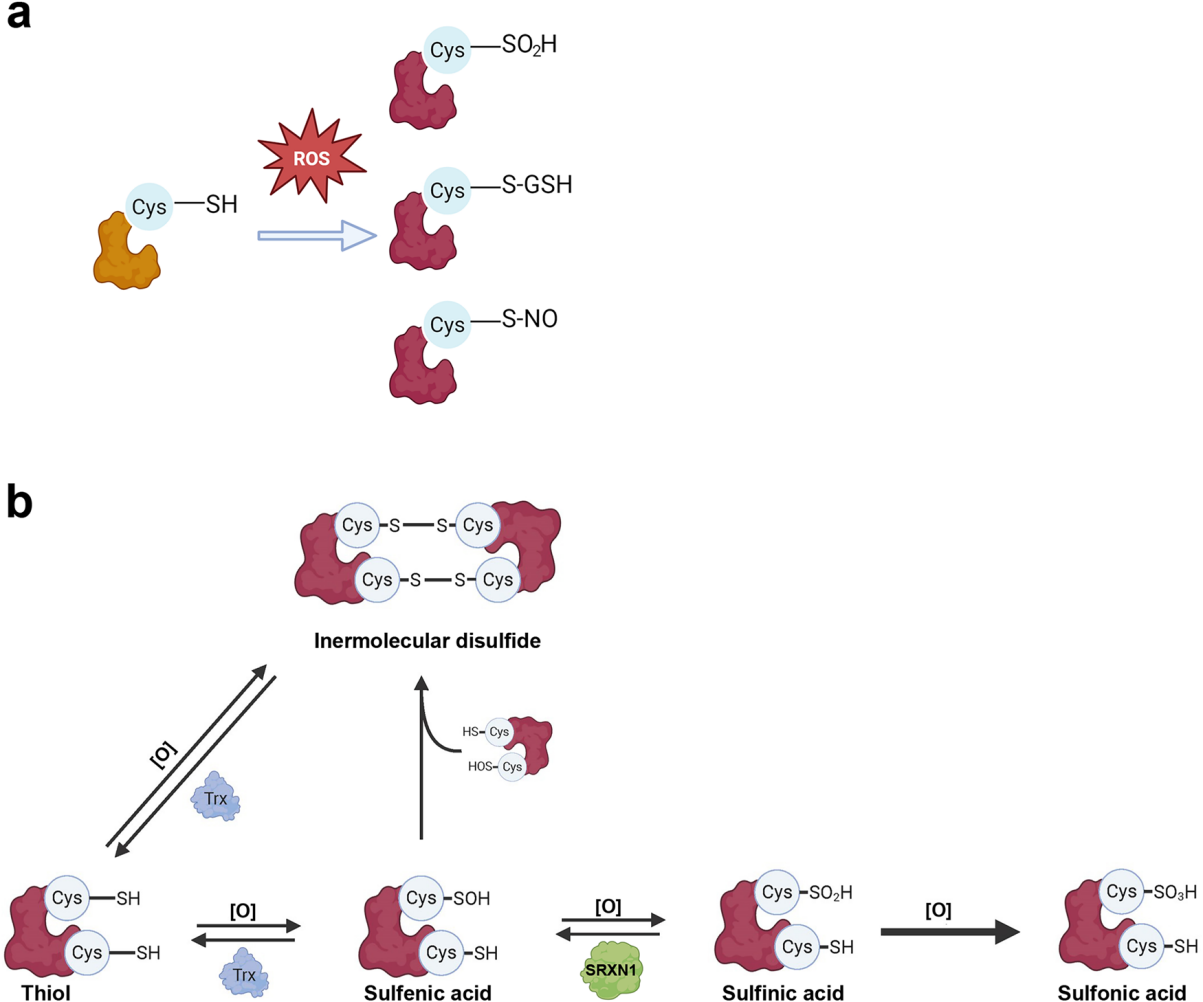
Cysteine oxidations and SRXN1-mediated desulfinylation. **a** ROS induce oxidative modifications of protein Cys residues, leading to various PTMs, such as Cys sulfinylation (–SO₂H), glutathionylation (–GSH) and nitrosylation (–NO). **b** Illustration of Cys sulfinylation and SRXN1-mediated desulfinylation. Cysteine residues are initially oxidized to sulfenic acid (Cys-SOH), which can be reduced back to thiol or further oxidized to sulfinic acid (Cys-SO₂H). Sulfinic acid can be reversed by SRXN1, while further oxidation to sulfonic acid (Cys-SO₃H) is irreversible. Cys-SOH can also undergo spontaneous disulfide bond formation. In addition, cysteine residues can form disulfide linkages through oxidation, which are reversible and can be reduced back to free thiols. These restorations of Cys residues help to maintain protein function and cellular redox homeostasis under oxidative stress conditions.

Oxidative stress, characterized by an imbalance between the production of reactive oxygen species (ROS) and the cellular antioxidant defenses, is a well-established contributor to the pathogenesis of a wide array of diseases, including cancers and noncancer diseases^1^. Central to this oxidative imbalance is the oxidation of cysteine (Cys) residues within proteins. Cys, a sulfur-containing amino acid, serves as a pivotal player in cellular redox regulation through its highly reactive thiol (–SH) group. This thiol group is subject to a variety of posttranslational modifications (PTMs), such as sulfinylation, glutathionylation and nitrosylation (Fig. 1a), which modulate protein functions, redox homeostasis and cellular responses to stress^2–4^. Dysregulation of these Cys-based PTMs has been implicated in disease progression, making them potential targets for therapeutic intervention.

Sulfiredoxin 1 (SRXN1) is an essential component of the cellular redox system, specifically tasked with reversing the hyperoxidation of peroxiredoxins (Prxs), thereby restoring their peroxidase activity^5^. By catalyzing the reduction of Cys sulfinic acid (Cys-SO_2_H) within Prxs (Fig. 1b), SRXN1 plays a critical role in protecting cells from excessive oxidative damage and maintaining redox balance. Beyond its interaction with Prxs, SRXN1 has been implicated in broader redox regulatory processes, including the modulation of signaling pathways involved in inflammation, apoptosis and cell survival through interacting with other target proteins^6^. Despite its established role in redox homeostasis, the function of SRXN1 in disease pathogenesis is only beginning to be elucidated. Recent evidence suggests that dysregulation of SRXN1 and its downstream effects on Cys oxidation may contribute to the progression of various pathologies, including cardiac inflammation^7,8^, brain damage^9^ and acute pancreatitis^10^, as well as its previously recognized roles in cancer^11^. Given its central role in regulating oxidative stress and its involvement in multiple disease processes, SRXN1 represents an attractive target for therapeutic intervention.

This Review aims to provide a comprehensive overview of SRXN1-mediated redox regulation of Cys oxidation, with a particular focus on its implications in liver disease mechanisms. We will explore the molecular mechanisms by which SRXN1 modulates redox signaling and discuss how dysregulation of this pathway contributes to the pathogenesis of liver diseases. Finally, we will evaluate the potential of targeting SRXN1 as a novel therapeutic strategy, considering both preclinical and emerging clinical evidence.

## Chemical characteristics of Cys in protein peptides

The thiol group in Cys is one of the most chemically reactive functional groups in proteins because it is prone to oxidation. Its unique properties allow it to form disulfide bonds (R–S–S–R) with another Cys residue within the same protein or between different proteins. Disulfide bond formation in Cys plays a pivotal role in the correct folding of proteins during biosynthesis, ensuring that proteins achieve their functional conformations^12^. Misfolded proteins containing aberrant disulfide bonds have been implicated in the development of liver diseases, including metabolic and alcohol-related liver disorders^13,14^.

In addition, cysteine residues play a critical role in regulating enzymatic activity due to their unique thiol groups, which confer high reactivity and enable diverse chemical interactions. These properties enable Cys to participate in redox regulation, covalent modifications and metal coordination, making it indispensable in enzymatic catalysis and cellular homeostasis. Cys residues in enzymes undergo oxidation to form disulfide linkages, which regulate the enzyme’s activity and ensure proper protein folding in the endoplasmic reticulum. These disulfide bonds can be reduced back to free thiols, allowing enzymes to function as redox switches in fluctuating oxidative environments^15^. Beyond disulfide bonds, Cys plays an essential role in metal-dependent enzymatic regulation by coordinating metal ions such as zinc and iron. Zinc-dependent enzymes, including alcohol dehydrogenase, utilize Cys residues to stabilize zinc ions within their active sites. This stabilization enhances catalytic efficiency in oxidation-reduction reactions^16^. Similarly, Cys residues coordinate iron–sulfur clusters ([Fe–S]) in enzymes, including aconitase, a citric acid cycle enzyme. These [Fe–S] clusters facilitate the isomerization of citrate to isocitrate, a critical step in cellular energy metabolism^17^.

More importantly, Cys residues in proteins undergo diverse PTMs that regulate protein activity, stability, localization and interactions. The thiol group in Cys contains an electron-rich sulfur atom, which, due to its *d*-orbital properties, can adopt multiple oxidation states. This chemical versatility enables a wide range of oxidative PTMs, leveraging the reactivity of the thiol group to facilitate dynamic responses to cellular and environmental changes. Understanding these modifications not only advances our knowledge of protein biology but also provides insights into therapeutic opportunities for diseases associated with dysregulated Cys modifications. Key Cys modifications, including S-sulfinylation, play distinct roles in cellular processes and signaling^18,19^.

## The historical discovery of SRXN1

Throughout the late twentieth century, research into oxidative stress mechanisms had largely focused on identifying key enzymatic players such as catalase, superoxide dismutase and glutathione peroxidase. These enzymes provided substantial knowledge about ROS detoxification. However, Prxs—another class of antioxidants—remained underexplored, particularly their regulation during hyperoxidation^20^. The discovery of SRXN1 represents an important advancement in the study of oxidative stress and redox biology.

Woo and colleagues demonstrated for the first time that Prxs, previously considered irreversibly inactivated upon hyperoxidation, could undergo a reversible reduction mechanism^21^. Although the exact enzymatic player responsible for this repair was not identified in the study, the work provided a critical conceptual framework for understanding reversible protein oxidation in redox signaling. These findings were pivotal in guiding subsequent research, including the discovery of SRXN1.

The discovery of SRXN1 was first reported by Biteau and colleagues in their study of redox biology. While examining oxidative stress responses in *Saccharomyces cerevisiae* (yeast), the authors identified SRXN1 as a key enzyme involved in reducing sulfinylated Prxs. SRXN1 catalyzes the ATP-dependent reduction of sulfinic acid in hyperoxidized Prx, a previously unknown reaction in biological systems^22^. Subsequent study uncovered its homologs in mammals, demonstrating its evolutionary conservation and universal biological importance. Human SRXN1 consists of 137 amino acids and has a molecular weight of approximately 14 kDa (ref. ^23^). Further studies revealed that the Cys residue at position 99 (Cys99) in SRXN1 is directly involved in the enzymatic mechanism that facilitates the reduction of sulfinic acid. Mutations or modifications at this Cys abolish the desulfinylation activity of SRXN1, impairing the cell’s ability to recover from oxidative stress^24,25^.

## Transcriptional regulation of *SRXN1*

### Regulation of *SRXN1* by nuclear factor erythroid 2-related factor 2 (Nrf2)

Nrf2 is a well-known antioxidative transcription factor that binds to the *cis*-regulatory antioxidant response element (ARE), regulating over 230 detoxification and antioxidant genes involved in the phase II response^26–28^. Hence, its dysregulation has been associated with a variety of oxidative stress-related and inflammatory diseases^29–31^. Activated by electrophilic or oxidative stimuli that raise ROS levels, the Nrf2 pathway protects cells from prolonged stress and DNA damage^32^. Nrf2 targets exert numerous cytoprotective effects, from the canonical redox homeostasis functions to DNA-damage repair, drug metabolism, and survival and proliferation, among others^33,34^.

Soriano and colleagues first identified *SRXN*1 as a potential Nrf2 target gene^29^. Subsequent in silico and experimental analyses of the *SRXN1* promoter showed a conserved ARE-like sequence across various mammals and nonhuman primates, along with three putative AREs in the human *SRXN1* promoter, supporting its Nrf2-dependent transcription^29,30^. After cloning the *SRXN1* gene promoter region and generating site-specific ARE-deletion luciferase reporter constructs containing each of the three identified ARE sites, Singh and colleagues reported that only ARE1, located at −228 bp from the transcription start site, was functional and involved in Nrf2-mediated transcription of the human *SRXN1* gene^30^. In addition, SRXN1 promoter activity decreased upon *Nrf2* knockdown, while treatment with 2,5-*t*-butylhydroquinone, an Nrf2 activator, significantly enhanced its activity^30^. Taken together, these findings conclusively identify *SRXN1* as an Nrf2 transcriptional target.

### Regulation of *SRXN1* by AP-1

Activating protein-1 (AP-1) refers to a group of homo- or heterodimeric transcription factors containing a basic leucine zipper domain, comprising four subfamilies: Fos, Jun, ATF and Maf^35^. Upon dimerization, these complexes bind to the *cis*-regulatory AP-1 binding site. Dimer-specific DNA binding preferences exist: Jun–Jun and Jun–Fos dimers favor the phorbol 12-*O*-tetradecanoate-13-acetate-responsive element (TRE) (TGACTCA), while Jun–ATF and ATF–ATF dimers preferentially bind to the cAMP-response element (CRE) (TGACGTCA)^36–38^.

Early evidence of SRXN1 as an AP-1 target gene was suggested using a candidate approach and bioinformatic analysis that revealed three predicted AP-1 binding sites within the *SRXN1* promoter region^38,39^. Consistently, AP-1 (c-Fos and c-Jun) overexpression significantly enhanced SRXN1 promoter activity, an effect diminished by site-directed mutations in the predicted AP-1 sites (TGAGTCA), confirming *SRXN1* as an AP-1 target^40^. Notably, one AP-1 binding site overlaps with the ARE sequence also regulated by Nrf2^29^. A series of luciferase reporter and chromatin immunoprecipitation–quantitative polymerase chain reaction assays found that mutations in either AP-1 binding site within the *SRXN1* promoter region led to a decrease in *SRXN1* expression, suggesting that both binding sites play a role in *SRXN1* gene regulation^41^. Moreover, mutations in the AP-1 binding site of the *SRXN1* promoter functioned to reduce Nrf2-mediated transcription, suggesting a complex interplay between AP-1- and Nrf2-mediated *SRXN1* expression^29^. Collectively, these findings establish that both AP-1 and Nrf2 coordinately regulate *SRXN1* expression, enabling its dynamic response to oxidative and electrophilic stress and limiting cytotoxic damage

## The role of SRXN1 in Cys modification and the methodologies to detect protein sulfinylation

Emerging evidence highlights SRXN1 as a critical enzyme that reverses oxidative PTMs of Cys residues, including sulfinylation, glutathionylation and nitrosylation. Notably, SRXN1 plays a predominant role in reducing Cys sulfinic acid (Cys-SO₂H) back to sulfenic acid (Cys-SOH), thereby restoring protein function. For instance, oxidative stress induces S-sulfinylation of the catalytic Cys residue in Prx1, inactivating the enzyme. SRXN1 reverses this modification, regenerating active Prx I and maintaining redox homeostasis^23^. Further study has indicated that SRXN1 repairs hyperoxidized 2-Cys Prx isoforms (Prx I to Prx IV) by reducing their Cys sulfinic acid groups in an ATP- and Mg²⁺-dependent manner. This activity is essential for preserving Prx antioxidant capacity and protecting cells from oxidative damage^24^.

Interestingly, Findlay and colleagues reported that SRXN1 is also a key enzyme catalyzing the removal of Cys-glutathione adducts^42^. S-glutathionylation is a reversible modification that involves the formation of a disulfide bond between a protein Cys residue and glutathione, serving as a crucial regulatory mechanism in cellular signaling under oxidative and nitrosative stress. Their findings revealed that *SRXN1* overexpression reduced glutathionylation levels and delayed the glutathionylation rate, suggesting its involvement in the deglutathionylation pathways. Notably, the protein tyrosine phosphatase 1B (PTP1B) was identified as a primary target of SRXN1-mediated deglutathionylation. SRXN1 contains a conserved Cys residue (Cys99) essential for its enzymatic activity, and mutation of Cys99 attenuated deglutathionylation, indicating that SRXN1 is critical for restoring PTP1B phosphatase function^42^.

SRXN1 can also regulate cellular redox homeostasis by regulating S-nitrosylation. Sunico and colleagues reported that SRXN1 removes the nitrosothiol (–SNO) group from Prx2^43^. Overexpression of SRXN1 reduced S-nitrosylated Prx2 (SNO-Prx2) levels, thereby protecting dopaminergic neurons and human-induced pluripotent stem cell-derived neurons from nitric oxide-induced oxidative stress. Furthermore, in vivo studies revealed significantly elevated SNO-Prx2 levels in the brains of both mice and humans with Parkinson’s disease, a condition characterized by nitrosative and oxidative stress. These findings highlight SRXN1’s denitrosylase activity and its neuroprotective potential^43^. Overall, SRXN1 serves as a key reductase that preserves protein function by modulating multiple oxidative PTMs.

Detecting Cys modifications is crucial for understanding protein function and redox biology. The progress in understanding sulfinylation has been hampered by the technical limitations of existing detection methods because oxidative PTMs of Cys are highly unstable^44^. While mass spectrometry remains the primary method for detecting these modifications, it is prone to artifacts and misinterpretations—particularly due to overlapping mass shifts with persulfides and the inherent instability of sulfenic acids, despite sulfinic acids being more stable^45^. Although antibodies have been developed against hyperoxidized forms of certain proteins, they lack specificity and are unsuitable for global profiling^46^. To address these challenges, Lo Conte and colleagues developed a chemical probe-based method for the selective detection of sulfinylation^45^. The authors designed NO-Bio, a C-nitroso-based probe that selectively reacts with sulfinic acids while minimizing interference from other cysteine oxidation states. NO-Bio incorporates a biotin handle, facilitating sensitive detection and enrichment of sulfinylated proteins^45^. This study addressed a critical gap in redox biology by providing a robust chemical tool for detecting protein sulfinylation. By overcoming the technical challenges associated with mass spectrometry artifacts and antibody specificity, this chemical probe method enhances our ability to study oxidative PTMs and their role in cellular signaling and disease pathology.

While earlier methods, such as NO-Bio, advanced sulfinylation research, they still had limitations in sensitivity and proteomic scalability. To overcome these challenges, Akter and colleagues developed DiaAlk, a clickable electrophilic diazene probe that enables highly selective detection and proteomic analysis of S-sulfinylated proteins^6^. This new approach outperforms NO-Bio by offering a more comprehensive and precise identification of sulfinylation sites and protein targets. Using this probe, chemical proteomics identified about 60 previously unrecognized protein substrates of SRXN1, expanding its known functional scope beyond Prxs.

This study represents a major advancement in redox proteomics, introducing DiaAlk as a superior tool for detecting and characterizing protein sulfinylation. By uncovering previously unknown SRXN1 substrates and demonstrating that sulfinylation is a regulated redox signal rather than an oxidative byproduct, the findings provide new insights into oxidative stress mechanisms and disease pathology.

## SRXN1 in liver diseases

### Acute liver injury

ROS play a crucial role in the pathophysiology of acute liver injury (ALI). Recently, Yuan and colleagues reported the protective function of dimethylarginine dimethylaminohydrolase 1 (DDAH1) in maintaining intracellular redox homeostasis, a process mediated by SRXN1. In HepG2 cells, hydrogen peroxide (H_2_O_2_) treatment suppressed *DDAH1* expression, and the loss of *DDAH1* exacerbated oxidative stress. Mechanistically, immunoprecipitation assays revealed a direct interaction between DDAH1 and SRXN1, which was enhanced under oxidative stress conditions. *SRXN1* depletion reduced cell viability and led to excessive ROS accumulation. Notably, *SRXN1* knockdown downregulated DDAH1 expression, whereas *SRXN1* overexpression counteracted H_2_O_2_-induced DDAH1 suppression, underscoring SRXN1’s essential role in sustaining *DDAH1* expression and activity under oxidative stress. In vivo, hepatic *DDAH1* deficiency aggravated oxidative stress and liver dysfunction in a carbon tetrachloride (CCl₄)-induced mouse model of ALI, which correlated with reduced SRXN1 expression. These findings suggest that DDAH1 facilitates SRXN1 recruitment to preserve redox balance, positioning SRXN1 as a potential therapeutic target for acute liver failure^47^.

Besides CCl₄, ALI can be induced by various other oxidative liver toxins, including acetaminophen, whose overdose is the leading cause of drug-induced ALI in clinical settings. Future studies are needed to comprehensively define the role of SRXN1 in different ALI contexts and to explore its potential as a therapeutic target for liver protection.

### Alcoholic liver disease

Chronic alcohol consumption enhances oxidative stress primarily through increased metabolism via cytochrome P450 2E1, generating ROS, acetaldehyde and harmful protein and DNA adducts^48,49^. These products activate inflammatory signaling pathways, leading to the production of proinflammatory mediators that drive hepatocyte apoptosis and necrosis. Bae and colleagues reported that chronic ethanol consumption induces hepatic *Srxn1* expression via Nrf2 activation, as indicated by elevated *Srxn1* levels in ethanol-fed wild-type (WT) mice but not in *Nrf2*-deficient mice. Among the 2-Cys Prxs, only Prx I underwent hyperoxidation in ethanol-fed WT mice. However, in *Srxn1*-deficient mice, both Prx I and Prx III exhibited hyperoxidation, whereas Prx II and Prx IV remained unaffected. Further analysis revealed that Prx I co-localizes with CYP2E1 on the cytosolic surface of the endoplasmic reticulum, placing it in close proximity to sites of ethanol-induced oxidative stress. In addition, ethanol-fed mice exhibited increased Srxn1 levels in the microsomal fraction, suggesting its translocation to regions where Prx I hyperoxidation occurs. Notably, *Srxn1* deficiency exacerbated ethanol-induced liver damage, underscoring its protective role against alcohol-mediated oxidative stress. These findings position SRXN1 as a promising therapeutic target for mitigating alcoholic liver disease (ALD)^50^.

### Hepatic stellate cell activation and liver fibrosis

Liver fibrosis is a progressive condition characterized by excessive liver scarring, with oxidative stress playing a key role in hepatic stellate cell (HSC) activation. HSC activation is central to the pathogenesis of liver fibrosis, as activated HSCs are the main source of fibrogenic cytokines and extracellular matrix proteins. Our group recently investigated the impact of protein sulfinylation and SRXN1 in liver fibrosis. Findings revealed that protein Cys sulfinylation was increased in activated HSCs and fibrotic livers of both mice and humans. *SRXN1* expression was also elevated, suggesting that the increase in SRXN1 might not be sufficient to fully counteract Cys sulfinylation in pathological conditions within HSCs^51^.

In HSC-specific *Srxn1*-knockout mice (*Srxn1*^ΔHSC^), loss of *Srxn1* enhanced HSC activation, increased protein sulfinylation and exacerbated liver fibrosis. Mechanistically, SRXN1 regulated phosphatase protein tyrosine phosphatase nonreceptor type 12 (PTPN12)^6,51^, a newly identified inhibitor of HSC activation, by preventing its sulfinylation at the Cys 164 residue. Overexpression of WT-*PTPN12* suppressed HSC activation, with an even stronger effect observed in the sulfinylation-resistant C164A mutant. *Ptpn12* overexpression using an AAV6-*Ptpn12* viral system alleviated CCl₄-induced liver fibrosis in *Srxn1*^fl/fl^ mice, but this protection was diminished in *Srxn1*^ΔHSC^ mice. Further analysis revealed that PTPN12 sulfinylation reduced its phosphatase activity and protein stability, thereby activating its novel substate, a profibrotic NOD-like receptor pyrin domain-containing protein 3 (NLRP3) by increasing pathologic tyrosine phosphorylation. SRXN1’s protective effects were abolished when NLRP3 was inhibited, whereas *PTPN12* overexpression suppressed NLRP3 activation—a suppression that was further enhanced when the sulfinylation-resistant C164A mutant was overexpressed. This study highlights the key role of Cys sulfinylation in the progression of liver fibrosis and identifies the SRXN1–PTPN12–NLRP3 axis as a critical regulator of liver fibrosis and a potential therapeutic target for intervention^51^.

### Hepatocellular carcinoma

Excessive ROS contribute to cellular damage through various mechanisms, including proto-oncogene activation, tumor suppressor gene inactivation, DNA repair impairment and lipid peroxidation. Recent studies have identified SRXN1 as a critical regulator of hepatocellular carcinoma (HCC) progression by neutralizing intracellular ROS and favoring cancer cell survival. A clinical study involving 269 patients with HCC revealed significantly elevated *SRXN1* expression in tumor tissues compared with adjacent normal liver tissues. Moreover, higher *SRXN1* levels were associated with worse survival outcomes and advanced clinicopathological features, including larger tumor size, higher tumor grade and increased metastasis^52^. These findings underscore the dual role of SRXN1 in maintaining redox homeostasis and driving tumor progression, highlighting its potential as a prognostic biomarker and therapeutic target in HCC.

Besides regulating redox homeostasis, SRXN1 may have promoted HCC cell proliferation through additional mechanisms. Transcriptome sequencing analysis revealed that *SRXN1* knockdown significantly altered the expression of genes involved in lysosome biogenesis. Given the essential role of lysosomes in autophagy, subsequent experiments confirmed that SRXN1 inhibition enhances autophagic flux and lysosomal biogenesis. Mechanistically, *SRXN1* knockdown induced the nuclear translocation of transcription factor EB (TFEB), a master regulator of autophagy and lysosomal function. Notably, treatment with the ROS scavenger *N*-acetylcysteine blocked both TFEB nuclear translocation and the increase in autophagic flux triggered by *SRXN1* silencing, demonstrating that these effects are also ROS dependent^53^. These findings highlight SRXN1’s role in regulating autophagy and lysosomal function through ROS-mediated mechanisms, further reinforcing its potential as a therapeutic target in HCC.

In addition, SRXN1 has been implicated in cancer cell migration and invasion. A recent study demonstrated that *SRXN1* knockdown significantly suppressed HCC cell migration and invasion by affecting the SRXN1 downstream target B cell translocation gene 2 (BTG2)^54^. Mechanistically, *SRXN1* depletion modulated intracellular ROS levels, which in turn influenced the migratory and invasive capabilities of HCC cells. Further analysis revealed that the ROS–p65–BTG2 signaling axis plays a pivotal role in regulating epithelial–mesenchymal transition, a key process driving cancer metastasis. In vivo studies using mouse subcutaneous xenograft and metastasis models further validated the tumor-promoting effect of SRXN1, demonstrating its ability to enhance both tumor growth and metastatic potential. Collectively, these findings establish SRXN1 as a critical protumorigenic and prometastatic factor in HCC^54^, underscoring its promise as a therapeutic target for limiting HCC progression and metastasis.

**Fig. 2:**
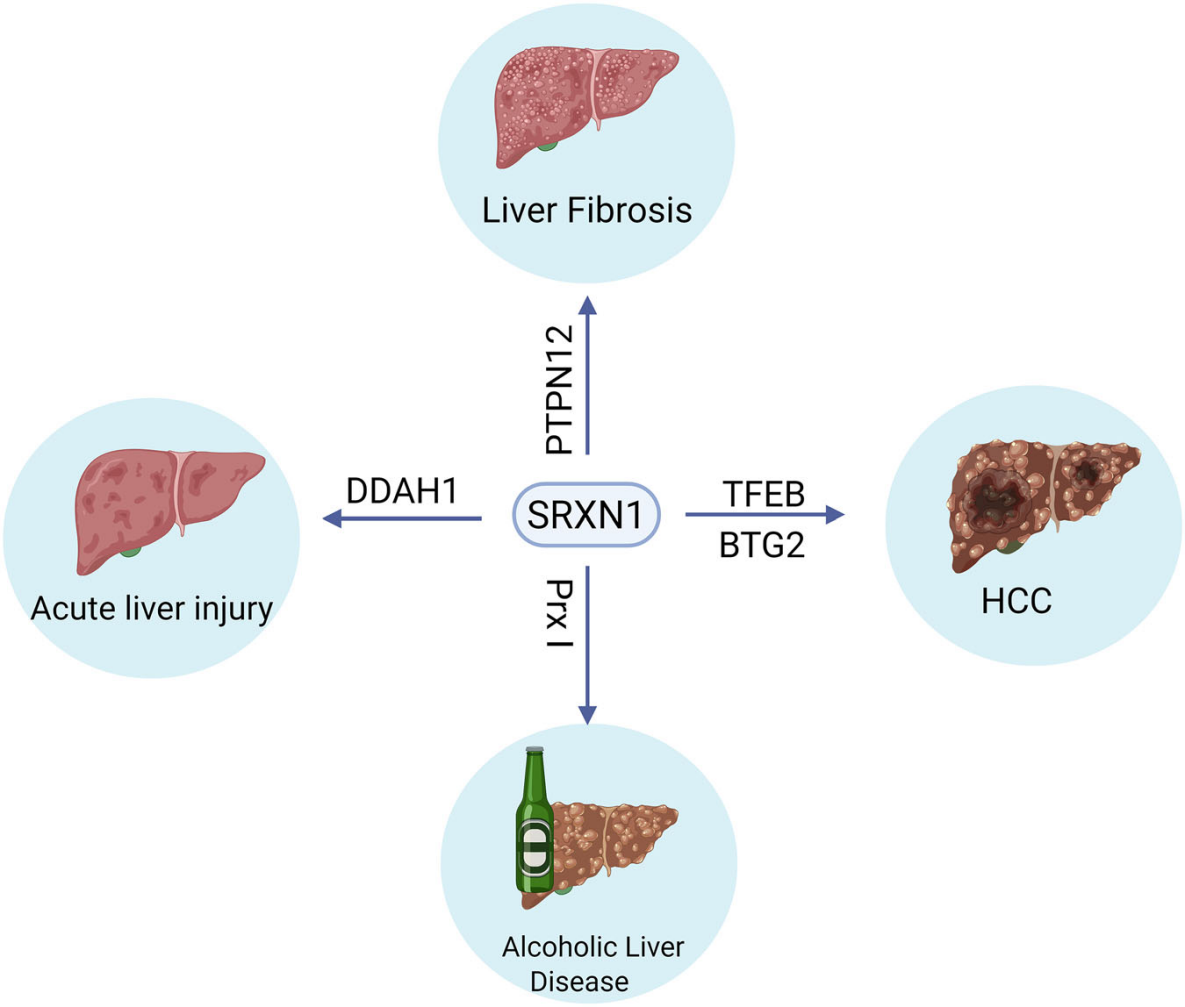
Summary of the role of SRXN1 in liver diseases and the key SRXN1 substrate proteins involved. Schematic overview of SRXN1’s role in diverse liver diseases, including ALI, ALD, liver fibrosis and HCC. The pathophysiological role of SRXN1 appears to be context dependent, exerting protective effects in ALI, ALD and liver fibrosis, while promoting tumorigenesis in HCC. The key substrate proteins that mediate the SRXN1 effects in each disease are labeled. DDAH1: Dimethylarginine Dimethylaminohydrolase 1; PTPN12: Protein Tyrosine Phosphatase, Non-Receptor Type 12; TFEB: Transcription Factor EB; BTG2: B-cell translocation gene 2; PrxⅠ: Peroxiredoxin I.

The currently known roles of SRXN1 in liver diseases are summarized in Table 1 and Fig. 2.

**Table 1:** Summary of SRXN1 functions in liver diseases.

Disease	Role of SRXN1	Key substrates or pathways	Therapeutic implications
**ALI**	Maintains redox homeostasis in CCl_4_ model of ALI	Interacts with DDAH1 to suppress H_2_O_2_-induced ROS	Potential target for redox-based protection in ALI
**ALD**	Attenuates ethanol-induced oxidative stress	Nrf2-induced SRXN1 expression and further protection against Prx hyperoxidation	SRXN1 activation mitigates ethanol-induced liver injury
**Liver fibrosis**	Suppresses HSC activation and liver fibrosis	SRXN1 suppresses sulfinylation of PTPN12 and inhibits NLRP3 activation	Targeting the SRXN1–PTPN12–NLRP3 axis ameliorates liver fibrosis
**HCC**	Promotes tumor growth, survival and metastasis	SRXN1 regulates autophagy and EMT through ROS-dependent pathways (via TFEB and BTG2)	SRXN1 serves as a prognostic biomarker and therapeutic target in HCC

### Limitations in current SRXN1 research models and translational considerations

The protective role of SRXN1 has been extensively investigated in preclinical models of ALI, ALD and hepatic fibrosis. However, a substantial proportion of these findings are derived from chemically induced rodent models, such as CCl₄ administration or ethanol exposure. Although these models have been instrumental in elucidating the mechanistic role of SRXN1 in oxidative stress-related liver injury, they possess inherent limitations in their translational relevance. For instance, CCl₄-induced hepatic fibrosis in rodents is known to resolve spontaneously following cessation of exposure, which contrasts with the persistent and often irreversible nature of fibrosis observed in human liver disease. In the context of ALD, interspecies differences in alcohol metabolism and cytochrome P450 isozyme expression may markedly affect ROS production and the kinetics of SRXN1 induction. These discrepancies highlight the need for cautious interpretation when extrapolating data from animal models to human pathology and underscore the importance of validating findings in clinically relevant systems.

In addition, although the role of SRXN1 has been extensively characterized in the context of hepatic pathophysiology, most existing studies have narrowly focused on liver-specific effects, without evaluating its potential systemic impact. This organ-restricted approach overlooks the fact that oxidative stress is a shared pathological driver across multiple organ systems—including the kidney, brain and cardiovascular tissues—raising the possibility that SRXN1 may participate in broader interorgan redox signaling networks. As such, the absence of data on how hepatic SRXN1 activity might influence or be influenced by redox dynamics in extrahepatic tissues represents a critical knowledge gap. Furthermore, the lack of longitudinal studies impedes a comprehensive understanding of how SRXN1 modulates disease trajectories or therapeutic responsiveness over time, particularly across different stages of liver disease. These limitations underscore the need for integrative and temporally resolved models that can delineate both tissue-specific and systemic roles of SRXN1 in oxidative stress-associated pathologies.

## SRXN1-based therapeutic approaches

While SRXN1 protects noncancerous cells in acute and chronic liver diseases, its overexpression in cancer cells, including HCC, promotes tumor survival, proliferation and resistance to oxidative stress-based therapies. As such, targeting SRXN1 with small-molecule inhibitors has emerged as a promising strategy to disrupt the antioxidant defenses of cancer cells and enhance ROS-induced cytotoxicity. Among the identified SRXN1 inhibitors, J14 and its derivative LMT-328 stand out for their promise in therapeutic applications^55^.

Recent studies highlight J14 as a competitive SRXN1 inhibitor that selectively disrupts the redox balance in cancer cells. J14 binds to the ATP-binding site of SRXN1, effectively preventing its catalytic activity. Molecular dynamics simulations have demonstrated that J14 exhibits a higher binding affinity to the ATP-binding site compared with ATP itself, forming stable interactions that impede the enzymatic repair of hyperoxidized Prxs^55^. The inhibition of SRXN1 amplifies intracellular ROS levels, leading to oxidative damage, mitochondrial dysfunction and apoptosis in cancer cells. Importantly, J14 shows selective cytotoxicity toward *SRXN1*-overexpressing cells, sparing normal cells that rely less on SRXN1 for their oxidative stress management. J14 also exhibited a synergistic antitumor effect with the kinase inhibitor sorafenib by promoting increased ROS levels and inducing autophagy^54,56^.

Building on the promising antitumor activity of J14, Kim and colleagues developed LMT-328, a structurally optimized derivative of J14 with enhanced potency. LMT-328 features additional hydrophobic and hydrogen bond interactions at the SRXN1 ATP-binding site, resulting in improved binding affinity and inhibitory activity^55^. This enhanced potency positions LMT-328 as a next-generation SRXN1 inhibitor with improved pharmacological properties for clinical development.

Cancer cells, particularly those in oxidative stress-prone environments such as the liver, depend heavily on SRXN1 to neutralize ROS generated by metabolic and environmental stressors^52^. By inhibiting SRXN1, J14 and LMT-328 render these cells vulnerable to oxidative damage, a vulnerability that can be exploited further in combination with ROS-inducing therapies such as chemotherapy, radiotherapy or photodynamic therapy^54^. Therefore, these inhibitors hold promises for overcoming resistance to existing cancer therapies, as SRXN1-mediated antioxidant defenses often underlie therapy resistance in tumors.

Despite the exciting potential of J14 and LMT-328, challenges remain to ensure the selective targeting of SRXN1 in cancer cells while preserving redox homeostasis in normal tissues. Biomarker-driven patient selection, based on SRXN1 expression levels, may enhance therapeutic efficacy and minimize off-target effects. Moreover, clinical trials must validate the safety, pharmacokinetics and long-term outcomes of J14 and LMT-328 in humans.

It is important to note that the roles of SRXN1 in pathophysiology are complex. In normal cells and under certain pathological conditions, such as inflammation or fibrosis^47,50,51^, SRXN1 exerts protective effects. In such cases, agents or strategies that enhance the expression and/or activity of SRXN1 may prove beneficial in the treatment of oxidative stress-associated diseases.

## Conclusions and future perspectives

As the understanding of redox biology continues to advance, SRXN1 has emerged as a critical regulator of cellular defense mechanisms against oxidative stress. One of the key functions of SRXN1 is cysteine desulfinylation of its target proteins. The pathophysiological significance of SRXN1 is context dependent. The activation SRXN1 is a double-edged sword, protecting normal cells while favoring the survival of cancer cells.

However, despite considerable progress, key questions remain regarding the specific roles of SRXN1 across various pathologies. Future research should aim to delineate the tissue-specific, cell-specific and disease-specific functions of SRXN1, particularly in diseases where oxidative stress plays a prominent role, such as cancers and acute or chronic inflammatory conditions. Another challenge is to identify and characterize the substrate proteins that are responsible for the biological effects of SRXN1.

The therapeutic potential of SRXN1 will become increasingly apparent. Preclinical studies suggest that manipulating SRXN1 activity can modulate oxidative stress levels, reduce cellular damage and improve disease outcomes. However, more research is needed to translate these findings into clinical applications. Developing pharmacological agents that specifically target SRXN1 and understanding its broader implications across different tissues and disease states will be crucial steps in advancing SRXN1-based therapies. These could involve small-molecule modulators that enhance SRXN1 activity in conditions where oxidative stress is excessive, or inhibitors in contexts where *SRXN1* overexpression contributes to disease progression, such as in certain cancers.

Nevertheless, several translational challenges must be addressed before SRXN1-targeted therapies can be successfully implemented in clinical settings. Off-target effects remain a significant concern due to the ubiquitous expression of SRXN1 and its essential role in preserving redox homeostasis in normal tissues. Achieving tumor selectivity is also challenging, as therapeutic agents must effectively discriminate between malignant and nonmalignant cells to avoid unintended toxicity. Furthermore, the intrinsic complexity and adaptability of redox signaling networks may trigger compensatory mechanisms or lead to therapeutic resistance. To overcome these obstacles, future strategies should prioritize the development of highly selective SRXN1 modulators, the optimization of tissue- or cell-specific delivery systems and the integration of pharmacodynamic biomarkers to monitor on-target effects and minimize systemic toxicity.

Furthermore, the potential of SRXN1 as a biomarker for disease diagnosis and prognosis warrants further investigation. Elevated or diminished *SRXN1* expression could serve as an indicator of oxidative stress levels and disease states, enabling earlier detection and more personalized treatment approaches. Further studies should also address the role of SRXN1 in modulating immune responses and inflammation, as these are increasingly recognized as critical contributors to chronic diseases. Given the central involvement of redox signaling in immune cell activation and inflammatory responses, SRXN1 may play a regulatory role in shaping immune cell behavior, particularly in macrophages. Macrophages are key mediators of inflammation and tissue remodeling, and their polarization states critically influence the tumor microenvironment. In the context of HCC, where an immunosuppressive microenvironment facilitates tumor progression, SRXN1 may contribute to immune evasion by modulating macrophage function or other immune cell subsets. Thus, inhibition of SRXN1 could potentially reprogram tumor-associated macrophages toward a proinflammatory, antitumor phenotype, thereby enhancing the efficacy of immunotherapies. Conversely, systemic inhibition of SRXN1 may impair redox homeostasis in immune cells, potentially dampening antitumor immunity. These dual possibilities underscore the importance of further mechanistic studies to delineate the impact of SRXN1 on immune cell function, with a focus on its role in cytokine production, macrophage polarization and immune checkpoint modulation. A clearer understanding of these interactions may uncover novel therapeutic opportunities at the intersection of redox biology and cancer immunotherapy.

In conclusion, SRXN1 represents a promising target for therapeutic intervention across a diverse range of pathologies driven by oxidative stress. By harnessing its redox regulatory properties, future therapies may offer new hope for patients suffering from chronic and debilitating diseases where current treatments remain insufficient. Continued exploration of SRXN1s biology will be essential to unlocking its full therapeutic potential.
